# Supplementary material for: Silver linings of the COVID-19 lockdown in New Zealand
Source: PLoS One. 2021 Apr 1;16(4):e0249678. doi: 10.1371/journal.pone.0249678 (PMC8016296; doi:10.1371/journal.pone.0249678)
Supplement: Attachment — Submitted filename: Response to Reviewers.docx [file pone.0249678.s001.docx]

**RESPONSE TO REVIEWERS**

**Academic Editor**

This was an interesting reading. I also conducted a similar study in my country (still under review), so it was interesting to compare results. However, I agree with the Reviewers that modifications are needed. Especially:

This study has a strange sampling technique for a qualitative study. One would expect that a qualitative study of this kind would adopt saturation sampling, namely researchers would collect data while performing first coding phases and stop when new themes cease to emerge. On the contrary it seems Authors here adopted a sampling typical of quantitative, questionnaire-based research. Why was it necessary to collect so many testimonies? Since so many data are available, and coding was performed on all of them, I would expect Authors to report descriptives/percentages of themes across the sample. In other words, since a notable sample is available, readers deserve to know how much a given theme was represented across the sample, which were prevalent or rare etc.

**Response:** *Thank you for seeking clarification of the sampling and subsequent analyses; we agree this aspect of the MS needs further development. We have modified the MS to explain that our study was not a traditional qualitative study, where the sample size is determined by data saturation or similar criteria (see p.6). Instead, we analysed open text responses that we collected as part of a larger quantitative survey study; we used qualitative thematic analysis to interpret these responses. The (large) sample size was thus determined by the sampling criteria used in our quantitative study. The richness of the many qualitative comments made it clear that we had a unique opportunity to provide new insights into how participants had experienced an unprecedented situation, and we have edited the manuscript to clarify our approach.*

*We agree that a traditional quantitative study would present data as you have suggested, However, in keeping with qualitative practice and follow the approach of other studies that have analysed open-ended comments, we have not quantified the themes we identified.*

For the same reason, and in accordance with Reviewers, more information and discussion could be added for table 1 information

**Response:** *We also agree that providing a clear description of the overall sample is important. We have clarified Table 1 but, for the reasons we noted above, we have not developed demographic profiles for each of the qualitative themes identified. We believe this approach aligns more clearly with thematic analysis and hope the clarifications we have made to introduce our study now support our qualitative orientation.*

Again in accordance with Reviewers, more example/excerpts could be added. To put it simply, I encourage Authors to make full use of the rich data they collected

**Response:** *More quotes have been added throughout the results section that illustrate each of the themes (see pages 12-16). Details of these changes can be seen in response to Reviewer 1’s comment #5.*

If you would like to make changes to your financial disclosure, please include your updated statement in your cover letter. Guidelines for resubmitting your figure files are available below the reviewer comments at the end of this letter.

If applicable, we recommend that you deposit your laboratory protocols in protocols.io to enhance the reproducibility of your results. Protocols.io assigns your protocol its own identifier (DOI) so that it can be cited independently in the future. For instructions see: [http://journals.plos.org/plosone/s/submission-guidelines#loc-laboratory-protocols](https://apc01.safelinks.protection.outlook.com/?url=http%3A%2F%2Fjournals.plos.org%2Fplosone%2Fs%2Fsubmission-guidelines%23loc-laboratory-protocols&data=04%7C01%7Cmatthew.jenkins%40otago.ac.nz%7Cf44cedb5690f4c50eeaa08d8a6496590%7C0225efc578fe4928b1579ef24809e9ba%7C1%7C0%7C637442181495044371%7CUnknown%7CTWFpbGZsb3d8eyJWIjoiMC4wLjAwMDAiLCJQIjoiV2luMzIiLCJBTiI6Ik1haWwiLCJXVCI6Mn0%3D%7C1000&sdata=2pjyPEr5jUP1PSBRLQxgPAJlqI2LEhHRQkZblttf7qE%3D&reserved=0)
Journal Requirements.

When submitting your revision, we need you to address these additional requirements.

 1. Please ensure that your manuscript meets PLOS ONE's style requirements, including those for file naming. The PLOS ONE style templates can be found at

<https://journals.plos.org/plosone/s/file?id=wjVg/PLOSOne_formatting_sample_main_body.pdf> and

<https://journals.plos.org/plosone/s/file?id=ba62/PLOSOne_formatting_sample_title_authors_affiliations.pdf>

2. Thank you for stating the following in the Acknowledgments Section of your manuscript:

'We would like to thank Dynata for their generous support of this research.'

We note that you have provided funding information that is not currently declared in your Funding Statement. However, funding information should not appear in the Acknowledgments section or other areas of your manuscript. We will only publish funding information present in the Funding Statement section of the online submission form.

a. Please remove any funding-related text from the manuscript and let us know how you would like to update your Funding Statement. Currently, your Funding Statement reads as follows:

'The authors received no specific funding for this work.'

**Response:** *The original statement is correct, we received no funding for this work. To clarify, we acknowledge and thank Dynata for their support in collecting data at such short notice and within a tight timeframe, not for financial support. We have edited this statement in the manuscript to clarify this (i.e., deleting the word ‘generous’, which may bring to mind financial support).*

b. Additionally, because some of your funding information pertains to [commercial funding//patents], we ask you to provide an updated Competing Interests statement, declaring all sources of commercial funding.

In your Competing Interests statement, please confirm that your commercial funding does not alter your adherence to PLOS ONE Editorial policies and criteria by including the following statement: "This does not alter our adherence to PLOS ONE policies on sharing data and materials.” as detailed online in our guide for authors  [http://journals.plos.org/plosone/s/competing-interests](about:blank).  If this statement is not true and your adherence to PLOS policies on sharing data and materials is altered, please explain how.

c. Please include the updated Competing Interests Statement and Funding Statement in your cover letter. We will change the online submission form on your behalf.

Please know it is PLOS ONE policy for corresponding authors to declare, on behalf of all authors, all potential competing interests for the purposes of transparency. PLOS defines a competing interest as anything that interferes with, or could reasonably be perceived as interfering with, the full and objective presentation, peer review, editorial decision-making, or publication of research or non-research articles submitted to one of the journals. Competing interests can be financial or non-financial, professional, or personal. Competing interests can arise in relationship to an organization or another person. Please follow this link to our website for more details on competing interests: [http://journals.plos.org/plosone/s/competing-interests](about:blank)

3. We note you have included a table to which you do not refer in the text of your manuscript. Please ensure that you refer to Table 2 in your text; if accepted, production will need this reference to link the reader to the Table.

**Response:** *Manuscript edited (this was a typo).*

**Reviewer #1:**

Thanks for the opportunity to read your manuscript. Without disclosing my identity, I'm a New Zealand social scientist living abroad (narrows it down to hundreds of people!) and I appreciated the opportunity to engage with this research about how New Zealanders experienced the COVID-19 Level 4 period.

Overall, I believe this manuscript makes an important contribution. Given the ongoing public health issues in creating a social consensus behind social distancing until vaccine take-up, I hope this will be published.

However, there are a couple of areas that need a little revision.

1) You could make more of the gender findings. It's unclear if we can re-interpret the figures in Table 1 as odds of finding silver-linings or not, but if they can the chance of women finding silver linings are significantly greater than for men : 684/345 = 1.98 (women) compared to 487/415 (men).

This finding is broadly consistent with other research in sociology during the pandemic that there are significant differences in reactions by gender. See, for example, this article from Germany, for which the research was conducted around the same time as your work: <https://www.tandfonline.com/doi/full/10.1080/14616696.2020.1808692>

**Response:** *As we noted above in our response to the AE, we have clarified the qualitative nature of our MS (see p.6). We have thus limited our comments on the sample sub-groups to avoid treating the data quantitatively. We have thus edited the table to present the overall sample demographic characteristics, rather breaking these details down according to whether participants provided information on silver linings or not.*

2) Related to this point, though less important, it might be useful to add a column to Table 1, displaying the composition of the New Zealand population for the same characteristics.

**Response:** *As noted above, although we believe this suggestion would be very helpful for a quantitative MS, we have followed qualitative tradition and described our overall sample.*

2a) In the panel for Ethnicity in Table 1, you might spell out New Zealand European.

We have made this change.

3) Under Environmental Re-creation, I wonder if you might use some of the quotes that mention birds and bird song to illustrate this theme (by the way, thanks for providing the data, this was an enjoyable part of this review)

**Response:** *Thank you for this suggestion. We have added the following quotes to these results (see p16): ‘Hearing birds sing’, ‘Seeing more native birds in our neighbourhood’, ‘Return of birds to cities’.*

4) I think that the theme you identify of agency in the "lockdown" is important, and undercuts the terminology of lockdown. This, to me, is the international significance of this paper. Just the basic findings that 2/3 of people found benefits in the lockdown, and the articulation of people coming together for the public health intervention, is evidence that the intervention succeeded because people could see the benefits. Clearly there was some limited enforcement of the limitations imposed under Level 4, but in large part this was achieved because people voluntarily did it.

**Response:** *Thank you for this comment. We agree that the question of agency is very important and appreciate the paradox that agency exists within an imposed lockdown. We have addressed this point in our discussion of self-determination theory, where we highlight the importance of agency under ‘lockdown’ restrictions. Please see page 19 where we note: “Although the COVID-19 lockdown restricted movement and reduced choices people could exert, providing a clear rationale for measures that reduced choice may have supported people’s perceived agency]”*

Around this point, you could cite and engage with the work on the leadership of Ardern.

McGuire, D., Cunningham, J. E., Reynolds, K., & Matthews-Smith, G. (2020). Beating the virus: an examination of the crisis communication approach taken by New Zealand Prime Minister Jacinda Ardern during the Covid-19 pandemic. Human resource development international, 23(4), 361-379.
Wilson, S. (2020). Pandemic leadership: Lessons from New Zealand’s approach to COVID-19. Leadership, 1742715020929151.

**Response:** *Thank you for the suggested citations. We agree that McGuire et al. have made an important contribution and have cited their work; please see reference (41) and our discussion on page 19. We have now reviewed Wilson’s commentary, and agree that this citation adds weight to our argument and have cited this, too.*

5) Once again, my thanks for providing the responses in a data repository. I think that it is important that before publication a wider range of quotes be used. Several quotes are used multiple to illustrate different themes. While quotes can clearly cross-cut themes, I think it would be stronger to have a range of different voices. The current situation suggests that there is not much depth to the responses, but the data doesn't bear this out: there are other quotes in the dataset that could replace duplicated quotes.

**Response:** *Thank you for the suggestion. More quotes have been added throughout the results section that illustrate each of the themes add richness to our interpretation and (see pages 12-16).*

*For example:*

*‘Being kinder to neighbours, grateful comments from the elderly who thought they had been forgotten and were lonely.’*

*‘People have become more attentive to each other in society’*

*‘It has made people interact with their closest family and friends, usually this would only happen on special occasions’*

*‘people smiling and greeting each other’*

*‘on social media there are always call outs from people who are offering to help families in need with food, and there are people who are just doing spontaneously nice things for others that you didn’t notice before.’*

*‘I think this break has been good for me, and for society. We do not need to go to shops and malls every day. We do not need to be full-time consumers. Shopping should be something we do sometimes, not all the time!’*

6) I hope that in the final dataset provided, you can include the thematic codes as well as the raw quotes.

**Response:** *Thank you for your interest. We have decided not to include the codebook with the data set because, considering the volume of responses, it is a very large document. However, we will make the codebook available to interested readers at their request.*

**Reviewer #2**:

This article presents a qualitative research on the benefits (silver linings) of COVID-19 lockdown in New Zealand, on people’s life.

In general I think that the paper and in particular the object of study is weak; authors may refer to positive psychology, not only to the specific area of post-traumatic growth, to improve the conceptualization of silver linings, that is the foundational concept of the study yet is little clarified. This should reflect in more specific research objective and interpretation of data.

The authors should improve the introduction as well, especially enhancing references on the epidemic and psychology (an impressive number of studies were published in the last months, especially on journals such as Personality and Individual Differences, Frontiers in Psychology, and PLOS ONE as well. I believe citations to these works should outnumber older citations on other epidemics or disasters).

At the same time, I think that the concept of post-traumatic growth is not very deepened and could be improved.

**Response:** *Thank you for encouraging us to consider expanding the conceptual framework underpinning our work. We have responded by reviewing additional studies on positive psychology, silver linings, and post-traumatic, and have included this material in the revised introduction. Please see pages 3-6, which now include more in-depth discussion around post-traumatic growth and benefit-finding, including up-to-date citations.*

Table 1 reports differences between participants who reported silver linings or not. Why are these differences relevant? This is not explored in discussion.

**Response:** *We agree that these details are less relevant to the qualitative approach that we have taken. We have clarified our orientation (please see our response to the AE above) and have modified Table 1 so that it provides a description of the sample rather than a profile of sub-groups within the sample.*

In methods, there is scarce explanation for the questions selected for the survey.
Sampling criteria are not specified, which is important since methodology (coding phases) seem to rule out saturation.

**Response:** *Thank you for seeking clarification of the questions used in the survey. We followed practices used in other surveys (e.g., Morphett et al., 2019) where general open-ended questions invite respondents to reflect on their experiences and to provide details of these. We have now referenced our approach to locate it within this established context.*

*We have also clarified how we obtained our data and thus the sampling strategy employed. Please see our response to the AE; we obtained the data from a larger quantitative survey study and our sampling criteria are those used in that study rather than the ‘traditional’ qualitative sampling criteria of data saturation. We have redeveloped the methods description to ensure the data source and sampling approach are now clearer.*

According to methods, apparently just one researcher coded the responses, while the others worked on the already coded data. Is this common procedure? Wouldn't it be better to include more coders and report inter-rater agreement?

**Response:** *In the current case, code development was an iterative process that involved three of the authors (MJ, JH, GJ). MJ developed initial codes, which JH then reviewed against her own coding. Discussions between MJ and JH refined these codes into an agreed set that GJ then reviewed. Further modifications were undertaken at this point and the codes used were agreed by all three coders. The process of code refinement was iterative and intensive rather than comparative, thus it is not appropriate to report inter-coder reliability scores. We have expanded the methods section to make this clearer.*

In my opinion, limitations and future research section could be stronger if authors would refer to other literature on psychological outcomes of the pandemic, and contribute to suggest the investigation of specific relevant psychological constructs and/or behaviors.

**Response:** *We have endeavoured to include up-to-date research on the psychological outcomes of COVID-19. However, this field is extremely dynamic, and we thus had to select a particular area of focus. Because New Zealand’s response has been internationally lauded and was among the most restrictive of those initially implemented, we believe reporting responses from the New Zealand population provides unique insights. We also note that studies on the positive psychological outcomes of COVID appear to have been undertaken (although an updated search has been undertaken since we wrote the manuscript).*

Finally, there are some typos in the manuscript. I suggest the authors to re-read the manuscript carefully.

**Response:** *Thank you for pointing these typos out. We have proof-read and edited the manuscript.*
