# Supplementary material for: Silver linings of the COVID-19 lockdown in New Zealand
Source: PLoS One. 2021 Apr 1;16(4):e0249678. doi: 10.1371/journal.pone.0249678 (PMC8016296; doi:10.1371/journal.pone.0249678)
Supplement: Attachment — Submitted filename: Response to Reviewers.docx [file pone.0249678.s002.docx]

**Letter of rebuttal for the manuscript: ‘Silver linings of the COVID-19 lockdown in Aotearoa New Zealand’**

***Editor’s comments***

While the effort in responding to review concerns was appreciable, and Reviewers are satisfied with the changes, I am still not convinced about how the sampling and data reporting aspect is managed in the manuscript. Since data were collected by an online source, demographics were collected, and data were coded within a large quantitative study, it is not clear why Authors are not able to report basic information on prevalence and demographics associated with the emerged themes. Authors report (just) one study as example with a similar approach, ref. 30, which I am not sure could be considered comparable because it has a smaller sample and a more specific topic (switching from smoking to vaping...).

In any case, most of the manual or methodological sources on thematic analysis I have seen say that prevalence of themes across the sample is an important information to report. Otherwise, we as readers are left with the impression that some interesting themes were chosen arbitrarily to present but we have no information on their distribution, internal variety and quantitative significance. Results risk to appear merely anecdotal and we are not sure whether the same information could have been found interviewing 500, 100 or 10 participants only.

If this is really impossible to do, Authors should at least:

- include thorough discussion of sampling and data reporting and justify their approach with proper methodological references

- detail this issue more in limitations and implications for future research

Furthermore, I was not able to see the research data on the link Authors provided ("website could not be reached"). Please make sure that data are available for consultation according to PLOS ONE's policies

**Authors’ responses**

We thank the editor for their comments. Please find our responses to each comment below.

- We purposely left out prevalence rates to avoid the risk of misleading readers about relative thematic significance. The number of times a theme appears in the data set does not necessarily indicate its significance as less represented themes may be just as important (sometimes even more important) as those more represented in the data (a key difference between quantitative and qualitative research). Further, we felt that reporting prevalence rates may imply a level of precision that cannot be supported by qualitative data, and that ‘presenting numbers can sometimes lead both the author and the reader to make inappropriate generalizations about the findings, albeit unconsciously’ (Neale, Miller & West, 2014, p.175).

Neale, J., Miller, P. and West, R. (2014) Editorial: Reporting quantitative information in qualitative research: guidance for authors and reviewers, *Addiction,* 109, 2, pp.175-176.

However, we understand that, for some people, adding numbers provides reassurance regarding scholarly rigour. Therefore, we have provided prevalence rates as percentages for the main themes (added to Table 2, p.9), though have included the caveat that prevalence rates do not necessarily equal relative significance (p.9), to reflect Neale et al.’s caution. We feel that clarifying the status of prevalence estimates is important in an open access journal article that publishes manuscripts with widely varying methods and attracts a more general audience that may be less familiar with differences between qualitative and quantitative research methodologies. We again acknowledge that the study does not follow traditional qualitative methodology, but we are confident that we took measures to ensure investigative rigour and hope that this compromise is sufficient.

- We agree that associating demographics to themes is an interesting aspect of this data to explore and something we believe should be investigated in future quantitative studies. However, to include this information in our current manuscript would require an in-depth and carefully considered quantitative analysis that moves beyond the scope and focus of the current paper (particularly given the word count limits). We acknowledge the editor’s suggestion but respectfully suggest this question is better explored in a future dedicated study, which we are currently considering.
- We apologise for the broken link to the raw data. This link was previously working (the data set had received positive comments by one of the reviewers). We have now re-uploaded the data and replaced the DOI.
- In response to Reviewer 1: *‘I would encourage you to provide the file with thematic codings as well as the raw quotes as part of the replication dataset.’* We have decided not to provide the thematic codebook. As stated in the previous response to reviewers, we do not see this as a critical inclusion for the purpose of publication, but reiterate that we will make the codebook available to interested readers at their request.
- Finally, please note that we also wish to change the title of the paper from ‘Silver linings of the COVID-19 lockdown in Aotearoa New Zealand’ to ‘Silver linings of the COVID-19 lockdown in New Zealand’. We have noted that Aotearoa (the Māori and indigenous name for New Zealand) may be mistaken for an area of New Zealand rather than the whole country. We feel that making it clear that this was a national study rather than a localised one is important.

Many thanks for your consideration, and we look forward to your response shortly.

Kind Regards

Matthew Jenkins
